# 648. Validation of a Synthetic Multiplexed External Control for Monitoring the Performance and Detection of Multiple Respiratory Viral and Bacterial Pathogens for a Point of Care Nucleic Acid Testing Panel

**DOI:** 10.1093/ofid/ofad500.711

**Published:** 2023-11-27

**Authors:** Nithya Sivaji, Emma Farrell, Tania R Spenlinhauer, Joan Gordon, Monica Cronin, Brad Graham, Jeffrey Bastar

**Affiliations:** Maine Molecular Quality Controls INC, Saco, Maine; Maine Molecular Quality Controls INC, Saco, Maine; Maine Molecular Quality Controls Inc., Saco, Maine; Maine Molecular Quality Controls INC, Saco, Maine; bioMérieux, Salt Lake City, Utah; bioMérieux, Salt Lake City, Utah; bioMérieux, Salt Lake City, Utah

## Abstract

**Background:**

Advancements in nucleic acid testing has enabled point of care testing solutions to provide clinicians with greatly improved time to diagnosis and patient care. However, complex technologies and regulatory requirements demand close monitoring of assay performance to accurately identify shifts, trends, and random errors to ensure accurate patient reporting. To support these requirements, MMQCI has developed a synthetic, easy-to-use, external control that simultaneously monitors the accurate identification of 15 respiratory pathogens (11 viral, 4 bacterial targets) detected with the BIOFIRE^®^ SPOTFIRE^®^ Respiratory (R) Panel.

**Methods:**

The multiplex control contains genome segments of all pathogens detected by the SPOTFIRE R Panel. Sequences were designed *in silico,* ligated into MMQCI vectors, and transformed to create stable frozen clone stocks. *In vitro* RNA transcripts were generated, quantified, and formulated in a proprietary matrix to stabilize and carry the RNA through the entire test process. Validation studies were conducted to demonstrate precision across multiple reagents, operators and sites. Failure-mode, shipping and stability studies were conducted to confirm robustness and reliability. Three unique lots of the SPOTFIRE RSP Positive and Negative controls were manufactured to include variability in key components and tested across 6 different sites, incorporating multiple reagent lots, operators and instruments. A total of 361 runs were performed across the positive and negative controls using the SPOTFIRE R Panel.

**Results:**

Of the 361 total runs across the 6 sites, 351 were valid and used for analysis. A total of 171/172 positive controls resulted in correct calls (99.3%), and all 179 negative controls were called correctly (100%). A regression analysis was performed to confirm real-time stability for 12 months when stored at temperatures ranging from 2^o^C to 25^o^C.

**Table 1.**
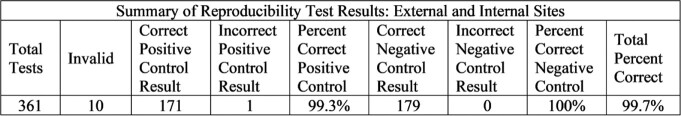
Summary of Reproducibility Test Results: External and Internal Site

**Conclusion:**

Validation studies demonstrated reproducibility, and overall precision and robustness of SPOTFIRE RSP Positive and Negative Controls. The SPOTFIRE RSP Positive and Negative Controls provide a stable, ease to use solution for training, verification and routine monitoring of the performance of the BIOFIRE^®^ SPOTFIRE^®^ Respiratory Solution.

**Disclosures:**

**Nithya Sivaji, MS**, Maine Molecular Quality Controls Inc.: employer **Emma Farrell, n/a**, Maine Molecular Quality Controls Inc.: employer **Tania R. Spenlinhauer, PhD**, Maine Molecular Quality Controls Inc.: employer **Joan Gordon, n/a**, Maine Molecular Quality Controls, Inc: President|Maine Molecular Quality Controls, Inc: Ownership Interest **Monica Cronin, n/a**, bioMerieux: Stocks/Bonds **Brad Graham, n/a**, bioMerieiuux: Employee **Jeffrey Bastar, n/a**, bioMerieux: Employee|bioMerieux: Stocks/Bonds

